# Real World Outcomes of Prescribed Ablative Dose Yttrium-90 Resin Selective Internal Radiation Therapy of Hepatocellular Carcinoma

**DOI:** 10.1055/s-0046-1825760

**Published:** 2026-07-15

**Authors:** Si Xuan Koo, You Kai Poh, Nathaniel Sheng Hua Too, Wei Ming Chua, Desmond Shi Wei Lim, Charles Xian Yang Goh, Winnie Wing Chuen Lam, Kelvin Siu Hoong Loke, Aaron Kian Ti Tong

**Affiliations:** 1 Department of Nuclear Medicine and Molecular Imaging, Singapore General Hospital, Singapore; 2 Division of Supportive and Palliative Care, National Cancer Centre Singapore, Singapore; 3 Duke-National University of Singapore Medical School, Singapore

**Keywords:** selective internal radiation therapy, transarterial radioembolization, hepatocellular carcinoma, liver cancer, Yttrium-90

## Abstract

**Objective:**

This retrospective single-center study sought to evaluate the efficacy and safety of predictive tumor-absorbed ablative doses of Yttrium-90 resin microsphere selective internal radiation therapy (SIRT) for hepatocellular carcinoma (HCC).

**Methods:**

We conducted a retrospective single-center study of patients with HCC treated at a tertiary referral center, who underwent Y-90 resin SIRT with ablative intent (defined as predictive tumor absorbed dose (TAD) of at least 190 Gy via planning single-photon emission computed tomography-CT dosimetry) between January 2016 and December 2023, evaluating radiological response and survival outcomes.

**Results:**

Among 68 patients, median TAD was 262.24 Gy (interquartile range 209.13 Gy-336.26 Gy). Median follow-up was 1,231 days (approximately 40.4 months). At 6 and 12 months post-treatment, objective response (OR) and disease control rates (DCR) in the target tumors were 87.5% and 96.9%, and 73.6% and 86.8%, respectively, assessed with modified Response Evaluation Criteria in Solid Tumors (mRECIST) on imaging.

Median target tumor time to progression (TTP) was not reached (95% CI: 785 days – not reached); portal venous invasion was an independent predictor for shorter TTP (HR 7.34, 95% CI 1.57–34.4, *p* = 0.011). Median progression-free survival (PFS) was 453.0 days (95% CI, 287–577). Barcelona Clinic Liver Cancer stages B/C (HR 2.87, 95% CI 1.49–5.53, *p* = 0.0016) and the absence of subsequent curative treatment (HR 2.13, 95% CI 1.10–4.12, *p* = 0.026) predicted for poorer PFS.

Median overall survival (OS) was 1,412 days (95% CI 596–NR). Absence of subsequent curative treatment predicted for poorer OS (HR 5.23, 95% CI 1.66–16.48, *p* = 0.005). Median OS for the 190 to 300 Gy group was 699 days (95% CI 531–2,244 days), and 1,446 days (95% CI 796 days – NR) for the TAD> 300 Gy group, though observed differences in OS between the groups were not statistically significant. Two patients had possible radiation-induced liver disease, two had post-embolization syndrome, and two had radiation-induced peptic ulcer disease. These six patients were primarily responsible for the grade 2 and 3 adverse events recorded.

**Conclusion:**

Y-90 resin SIRT with predictive TAD of at least 190 Gy in HCC can achieve favorable 1-year target tumor disease control and a median OS of 46.4 months. Absence of subsequent curative treatment independently predicted inferior progression-free and overall survival. Among plausible reasons for the absence of a clear dose–response relationship, TAD substantially higher than 190 Gy may be required for consistent ablative efficacy.

## Introduction

Selective internal radiation therapy (SIRT) with Yttrium-90 (Y-90) microspheres, otherwise known as transarterial radioembolization, is a crucial tool in the armamentarium for treating hepatocellular carcinoma (HCC). Besides being the next-line treatment option for patients when Barcelona Clinic Liver Cancer staging system (BCLC) B tumors are ineligible for transarterial chemoembolization (TACE), or when BCLC C tumors are unsuitable for systemic therapy, it is also an excellent option for bridging to resection tumors when surgery is not immediately feasible, or for downstaging to transplant. Two types of Y-90 microspheres are used clinically – resin and glass. Despite differences in particle size, activity per microsphere, and number of injected microspheres, there have been no proven significant survival differences between them. 1 The LEGACY trial showed that glass Y-90 microspheres can be used to safely and efficaciously achieve local tumor control with durable response in unresectable solitary HCC ≤8 cm, 2 and the United States Food and Drug Administration has since approved glass Y-90 microspheres for the treatment of unresectable HCC in patients with preserved liver function, including the use of radiation segmentectomy. However, limited data exist on the optimal ablative dose of resin Y-90 microspheres that could achieve prolonged duration of response in patients with HCC.

## Methods

### Yttrium-90 SIRT Procedure

Patients with HCC tumors deemed suitable for Y-90 SIRT, through multidisciplinary discussion and consensus between medical oncologist, hepatologist, liver surgeon (or at least one of these), and nuclear medicine physician, would then undergo a planning hepatic angiography and intra-arterial injection of technetium-99m-labelled macroaggregated albumin (Tc-99m MAA) into targeted vascular territories supplying the tumor or tumors (henceforth referred to collectively as “target tumor” regardless of multiplicity). Both planar and single-photon emission computed tomography-CT (SPECT-CT) were then performed on the same day, within hours of the angiography and Tc-99m MAA injection. In general, patients with significant extrahepatic Tc-99m MAA localization, such as, gastrointestinal, or pulmonary localization (defined as liver–lung shunt percentage greater than 20% or resulting in a planned radiation dose to the lungs of more than 20 Gy), seen on the Tc-99m MAA scans were contraindicated for Y-90 SIRT.

Eligible patients subsequently received Y-90 SIRT using resin microspheres (SIR-Spheres, Sirtex Medical, United States) within 4 weeks of the Tc-99m MAA study.

### Patient Selection

Consecutive adult patients with HCC (diagnosed according to the American Association for the Study of Liver Diseases [AASLD] criteria 3 ) who had undergone SIRT with resin microspheres (henceforth referred to as “Y-90 SIRT”) at a tertiary hospital between January 01, 2016 and December 31 ^st^ , 2023 with ablative intent, defined as a documented predictive weighted average tumor absorbed dose (TAD) of at least 190 Gy, were enrolled. The dose threshold of ≥190 Gy was selected based on seminal radiopathologic glass-microsphere radiation segmentectomy studies 4 5 6 demonstrating strong correlations between absorbed dose and complete pathologic necrosis (CPN) in explants. At the time of study design, radiopathologic evidence defining dose thresholds associated with CPN in resin-microsphere cohorts was sparse, thereby necessitating extrapolation from the more mature glass-microsphere evidence base.

Calculation of the predictive TAD was derived from Tc-99m MAA SPECT/CT using the partition model and Medical Internal Radiation Dose schema, with input of tracer counts and volumes obtained from a volume of interest encompassing the tumor, non-tumorous liver, and lung (tricompartment model) or the perfused volume and lung (bicompartment model). Regions of interest were drawn over the hepatic tumors (including portal vein tumor thrombi), non-target liver parenchyma and lungs, supervised by experienced nuclear medicine physicians using OsiriX DICOM viewer (Pixmeo, Geneva). Weighted average TAD was used for the calculation in multipartition models.

All patients who fulfilled the above criteria were included in the overall cohort. Clinical information was extracted from Sunrise Clinical Manager (Allscripts Health Solutions INC, Chicago, Illinois, United States) and recorded in a prospectively maintained research database. The data, updated up to May 15, 2025, were submitted to the hospital's Health Sciences Research Unit for de-identification by an appointed trusted third party prior to subsequent analysis. Baseline patient and tumor characteristics, dates of last follow-up, progression or death, and modified Response Evaluation Criteria in Solid Tumors (mRECIST) radiologic response after treatment were recorded.

### Outcome Definitions

Tumor response was assessed using mRECIST criteria for HCC 7 on contrast-enhanced CT/MRI at 6 and 12 months after Y-90 SIRT. Two independent radiologists independently assigned target tumor and overall patient mRECIST responses (per-patient aggregate disease, including new intra- and extrahepatic lesions). In cases of discordance, a consensus assessment was reached through joint review by both readers. Response categories were complete response (CR), partial response (PR), stable disease (SD), or progression of disease (PD).

*Objective response rate (ORR)* was defined as the summed proportion of CR and PR, and *disease control rate (DCR)* as the summed proportion of CR, PR, and SD.

Patients were included in target tumor mRECIST analyses if the tumor remained evaluable, even if other oncologic treatments were given; however, definitive procedures (resection or transplantation) led to exclusion from further target tumor analyses. This reflects real-world practice, where additional treatments may be initiated in the absence of formal mRECIST progression. Patient-level mRECIST was assessed at 6 and 12 months regardless of intervening treatments. Patients who died, were lost to follow-up, or lacked imaging within 2 months of each timepoint were excluded from response analyses.

*Target tumor time to progression (TTP)* was defined as time from Y-90 SIRT to progression of the treated tumor by localized mRECIST. Patients were censored at subsequent hepatic interventions (TACE, ablation, resection, transplantation) if no progression had been documented, or at last follow-up/death if progression had not occurred.

*Progression-free survival (PFS)* was time from Y-90 SIRT to progression at any site found on imaging (intra- or extrahepatic), or death, whichever occurred first. Patients without either disease progression or death were censored at last follow-up. All patients with at least one evaluable post-treatment imaging study were included, even if the Y-90 target tumor was resected.

*Overall survival (OS)* was defined as time from Y-90 SIRT to death from any cause. Vital status was determined from medical records and the national death registry. Patients without documented death were censored at their last follow-up if seen within 6 months of study closure.

Loss to follow-up was defined as the absence of contact within 6 months prior to censoring, and these patients censored at their last known contact for all outcome analyses.

### Statistical Analysis

Patient demographics were summarized with descriptive statistics. Patient selection and the number of patients included in each time-to-event outcome analysis (target tumor TTP, PFS, OS) are summarized in the study flow diagram included in the Supplementary Appendix ( Supplementary Fig. S1 , available in the online version only).

Univariable and multivariable Cox proportional-hazard regression models were performed to identify predictors for each of the time-to-event endpoints (target tumor TTP, PFS, OS). Variables with *p* -value <0.10 on univariable analysis and clinically relevant covariates were entered into the multivariable Cox regression model. Results of all univariable analyses are provided in the Supplementary Appendix ( Supplementary Tables S1–S3 , available in the online version only), while results of the multivariable analyses are presented in Tables 1 to 3 . Kaplan–Meier curves are shown for variables independently associated with outcomes on multivariable analysis.

**Table 1:** Multivariable Cox regression for target tumor time to progression

Variable	Adjusted HR (95% CI)	*p* -Value
Size ≥8 cm vs. Size <8 cm	0.47 (0.09–2.34)	0.354
Portal vein invasion vs. no portal vein invasion	7.34 (1.57–34.4)	0.011
No subsequent curative treatment vs. subsequent curative treatment	3.30 (0.41–26.3)	0.269
No previous local treatment vs. previous local treatment	0.21 (0.02–2.48)	0.218
TAD >300 Gy vs. TAD 190–300 Gy	2.46 (0.70–8.65)	0.159

Abbreviation: CI, confidence interval; HR, hazard ratio; TAD, tumor absorbed dose.

**Table 2:** Multivariable Cox regression for overall progression-free survival

Variable	Adjusted HR (95% CI)	*p* -Value
Age >70 vs. age ≤70	0.90 (0.51–1.59)	0.726
Female vs. male	1.77 (0.95–3.32)	0.073
BCLC B/C vs. BCLC A	2.87 (1.49–5.53)	0.0016
No subsequent curative treatment vs. subsequent curative treatment	2.13 (1.10–4.12)	0.026
TAD >300 Gy vs. TAD 190–300 Gy	1.02 (0.58–1.79)	0.950
Bilobar liver disease vs. unilobar disease	1.14 (0.51–2.57)	0.746

Abbreviations: BCLC, Barcelona Clinic Liver Cancer staging system; CI, confidence interval; HR, hazard ratio; TAD, tumor absorbed dose.

**Table 3:** Multivariable Cox regression for overall survival

Variable	Adjusted HR (95% CI)	*p* -Value
Age >70 vs. age ≤70	1.04 (0.50–2.17)	0.922
Female vs. male	1.76 (0.78–3.98)	0.174
BCLC B/C vs. BCLC A	2.24 (0.92–5.46)	0.077
No subsequent curative treatment vs. subsequent curative treatment	5.23 (1.66–16.48)	0.005
TAD >300 Gy vs. TAD 190–300 Gy	0.51 (0.23–1.14)	0.101
Bilobar liver disease vs. unilobar disease	2.25 (0.81–6.27)	0.121

Abbreviations: BCLC, Barcelona Clinic Liver Cancer staging system; CI, confidence interval; HR, hazard ratio; TAD, tumor absorbed dose.

Clinical and laboratory adverse events within 6 months after the Y-90 treatment were graded using Common Terminology Criteria for Adverse Events, version 5.0. 8

## Results

A total of 68 patients met the dosimetric criterion of a weighted average TAD ≥190 Gy. OS was evaluable in all 68 patients. For the other outcomes, which required at least one post-treatment imaging study, 67 patients were assessable; one patient had no follow-up imaging after Y-90 treatment and was therefore excluded from these analyses.

Median follow-up was 1,231 days (approximately 40.4 months), calculated using the reverse Kaplan–Meier method which accounts for censoring of patients due to death. The baseline patient and tumor characteristics are listed in Table 4 . The median age of the cohort was 72.5 years (range 65.8–79.0 years), 52 out of 68 (76.5%) patients were male. Almost half (48.5%) the patient cohort had viral hepatitis B/C as their cirrhosis etiology, while four (5.9%) patients did not have cirrhosis.

**Table 4:** Patient and disease baseline characteristics

		All	TAD 190-300 Gy	TAD >300 Gy	*p* -Value b
Patients, *n*		68	44	24	
TAD (Gy) a	Median (Q1, Q3)	262.24 (209.13–336.26)	226.2 (201.5–254.4)	407.5 (332.8–470.1)	
	Range	190–1500	190–295.7	300.5–1500.0	
Age (years) a	Median (Q1, Q3)	72.50 (65.75–79.00)	73.5 (66.0–79.3)	71.0 (62.5–77.3)	0.11
	Range	38–89	40–89	38–82	
Sex	Male	52 (76)	34 (77)	18 (75)	0.79
	Female	16 (24)	10 (23)	6 (25)	
ECOG	0–1	57 (84)	36 (82)	21 (88)	0.83
	2–3	6 (9)	4 (9)	2 (8)	
	Not stated	5 (7)	4 (9)	1 (4)	
Cirrhosis etiology	Hepatitis B/C	33 (49)	23 (52)	10 (42)	0.21
	NASH	23 (34)	12 (27)	11 (46)	
	Alcoholic	2 (3)	1 (2)	1 (4)	
	Cryptogenic	4 (6)	3 (7)	1 (4)	
	Not stated	2 (3)	1 (2)	1 (4)	
	No cirrhosis	4 (6)	4 (9)	0	
Child-Pugh class	A	55 (81)	35 (80)	20 (83)	0.93
	B	9 (13)	6 (14)	3 (13)	
	Not applicable	4 (6)	3 (7)	1 (4)	
BCLC stage	A	41 (60)	27 (61)	14 (58)	0.83
	B	16 (24)	9 (20)	7 (29)	
	C	11 (16)	8 (18)	3 (13)	
Target tumor size	<8 cm	49 (72)	30 (68)	19 (79)	0.32
	≥8 cm	19 (28)	14 (32)	5 (21)	
Liver disease distribution	Unilobar	55 (81)	35 (80)	20 (83)	0.75
	Bilobar	13 (19)	9 (20)	4 (17)	
Liver tumor burden	Solitary	45 (66)	31 (70)	14 (58)	0.11
	2–5 tumors	19 (28)	10 (23)	9 (38)	
	>5 tumors	4 (6)	3 (7)	1 (4)	
Prior local treatment to target tumor		9 (13)	8 (18)	1 (4)	0.048
Prior systemic treatment		10 (15)	6 (14)	4 (17)	0.56
Subsequent local treatments (any; includes curative)		28 (41)	19 (43)	9 (38)	0.82
Subsequent curative treatment		17 (25)	12 (27)	5 (21)	0.78
Subsequent systemic treatment		35 (52)	20 (45)	15 (63)	0.24
Target tumor location relative to prior disease site c	No previous disease	50 (74)	30 (68)	20 (83)	0.07
	Separate	5 (7)	2 (5)	3 (13)	
	Adjacent	8 (12)	8 (18)	0	
	Within	5 (7)	4 (9)	1 (4)	
Portal venous invasion	None	59 (87)	38 (86)	21 (88)	0.17
	Branch	4 (6)	4 (9)	0	
	Main/beyond (SMV)	5 (7)	2 (5)	3 (13)	
Systemic venous involvement	None	64 (94)	40 (91)	24 (100)	0.37
	Hepatic vein	1 (2)	1 (2)	0	
	Hepatic vein, IVC, beyond	3 (4)	3 (7)	0	
Dosimetry method	Bicompartmental	9 (13)	5 (11)	4 (17)	0.72
	Tricompartmental	59 (87)	39 (89)	20 (83)	

Abbreviation: TAD, tumor absorbed dose.

a Continuous variables represented as median (IQR), all other values in this table are *n* (%).

b Patients whose target tumor received TAD between 190 and 300 Gy compared to patients whose target tumor received TAD greater than 300 Gy, using Fishers exact test for categorical data and non-parametric Mann–Whitney U test for continuous data.

c Prior disease refers to previously treated or documented tumor involvement at the target tumor location before Y-90 treatment.

Forty-one (60%) patients' target tumors were BCLC A, 16 (24%) were BCLC B, and 11 (16%) were BCLC C. Median tumor size was 6.5 cm (interquartile range [IQR] 4.78–8.53 cm) and the largest size of tumor targeted was 12.7 cm, involving three hepatic segments. Thirteen (19%) patients had bilobar hepatic disease (including previously treated tumors, or tumors being treated with other modalities), 23 (34%) patients had two or more tumor. Nine (13%) patients had portal venous invasion (PVI), out of which five patients had main or beyond PVI, and four patients had branch PVI. Nine (13%) patients had prior local treatment to the target tumor. Seventeen (25%) patients had subsequent curative treatment (percutaneous ablation, surgical resection or liver transplant) to the target tumors.

Nine (13%) patients had bicompartmental dosimetric planning, and 59 (87%) patients had tricompartment dosimetry data by MIRD calculation. Mean TAD was 311.5 Gy, median dose was 262.24 Gy (IQR 209.13–336.26 Gy). Forty-four (65%) patients had TAD of 190 to 300 Gy to their target tumor, and 24 (35%) patients had TAD above 300 Gy.

### Target Tumor and Patient Response Evaluations Based on mRECIST

#### Target Tumor mRECIST Response

##### At 6 Months Post-Treatment

Four patients were excluded from the evaluation of target tumor mRECIST responses at the 6-month timepoint (two had no imaging available, one had resection of the tumor about 3 months post-Y90 treatment, and one died). Among the 64 evaluable patients, CR of the target tumor was achieved in 17 (26.6%) patients, PR in 39 (60.9%) patients, SD in six (9.4%) patients, and two (3.1%) patients showed PD in the treated tumor. Target tumor ORR was 87.5%, target tumor DCR was 96.9%.

##### At 12 Months Post-Treatment

Fifteen patients were excluded from the evaluation of target tumor mRECIST responses at the 12-month time point (six patients had no imaging available for review at that time point, five had died by then, three had undergone tumor resection, and one was lost to follow-up). Among the 53 evaluable patients, the categories were as follows: CR, 23 (43.4%); PR, 16 (30.2%); SD, 7 (13.2%); and PD, 7 (13.2%). The target tumor ORR was 73.6%, and the target tumor DCR was 86.8%.

#### Overall Patient mRECIST Response

##### At 6 Months Post-Treatment

Three patients were excluded from overall patient mRECIST response evaluation at the 6-month time point (two had no imaging available, and one had died). Among the 65 evaluable patients, the categories were as follows: PR, 33 (50.8%); PD, 17 (26.2%); CR, 12 (18.5%); and SD, 3 (4.6%). The overall ORR was 69.2%, and the overall DCR was 73.8%.

##### At 12 Months Post-Treatment

Eleven patients were excluded from overall patient mRECIST response evaluation at the 12-month time point (four patients had no imaging available for review by that time, five patients had died by then, and two were lost to follow-up). Among the 57 evaluable patients, the categories were as follows: PD, 22 (38.6%); CR, 20 (35.1%); PR, 11 (19.3%); and SD, 4 (7.0%). The overall ORR was 54.4%, and the overall DCR was 61.4%.

No statistically significant median dose difference was found between responders and non-responders for OR and DCRs at 6 and 12 months, on a per-target tumor and per-patient basis. In addition, our analysis could not define an “optimal” dose threshold to predict OR or disease control at 6 and 12 months, on a per-target tumor and per-patient basis.

##### Target Tumor Time to Progression

Median time to progression (TTP) for the target tumor was not reached (95% CI: 785 days–NR; IQR: 639 days–NR). Sixty-seven patients were included in the analysis, with 17 actual progression events and 50 censored observations. Patients were censored either at the time tumors were subjected to surgery or other local treatments (primarily TACE) before demonstrating true PD (i.e., only SD or PR up till time of the other treatments) ( *n* = 15), or at the time of last follow-up or death if the tumors had not progressed by those respective points ( *n* = 35).

**Fig. 1:**
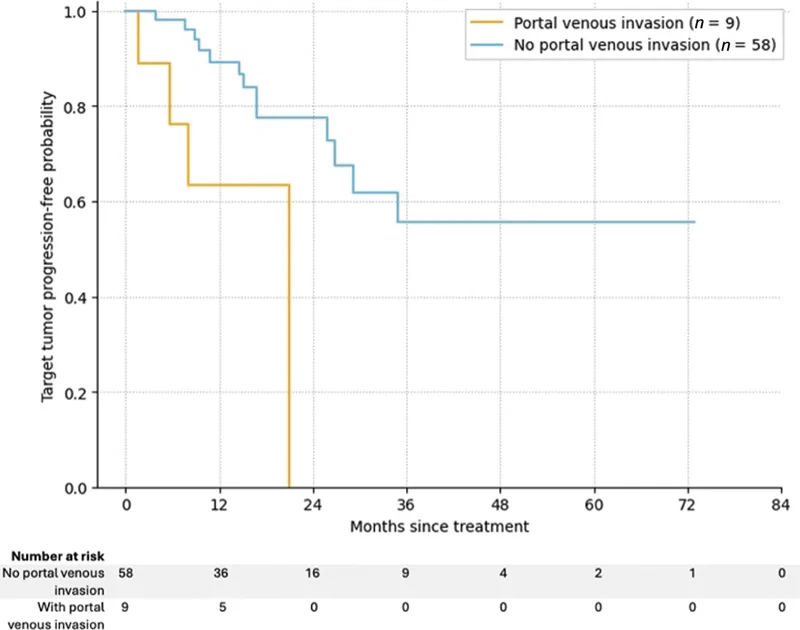
Kaplan–Meier curves of target tumor time to progression stratified by portal venous invasion.

Portal venous invasion was independently associated with shorter target tumor TTP ( Fig. 1 ).

Supplementary Table S1 (available in the online version only) in the Supplementary Appendix details the univariable Cox regression, while Table 1 details the multivariable Cox regression for target TTP.

##### Overall Progression-Free Survival

A total of 67 patients were evaluated for overall PFS, with one patient excluded because no evaluable imaging was available post-Y90 treatment. Median PFS was 453.0 days (95% CI, 287–577; IQR: 193–848 days).

Fifty-eight of the 67 patients showed progression or died during follow-up. Eight patients were censored because they had not shown evidence of PD by the time of last follow-up, while one patient with new hepatic lesions of indeterminate origin (e.g., possible metastatic disease from a secondary pancreatic cancer that was diagnosed 20 months after the Y90 RE for HCC) was censored at the time of lesion appearance, as progression from HCC could not be confidently determined.

**Fig. 2:**
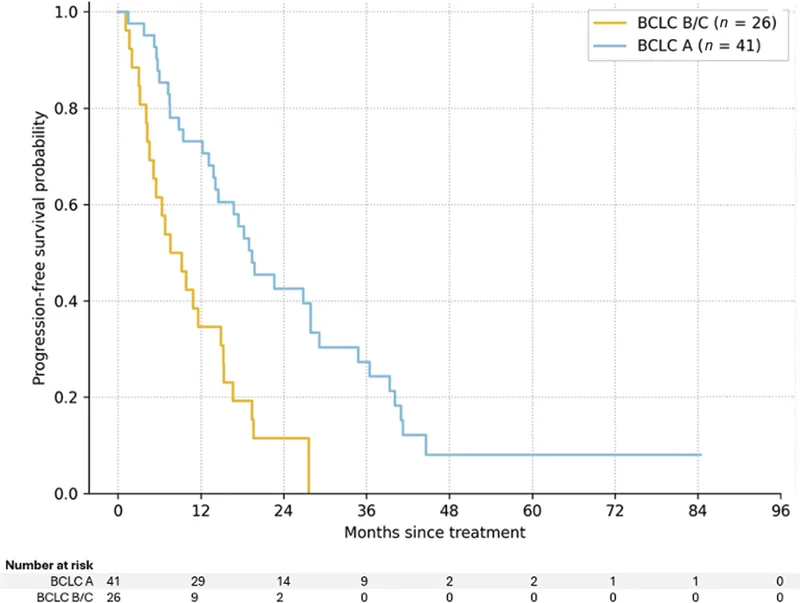
Kaplan–Meier curves of overall progression-free survival stratified by BCLC stage. BCLC, Barcelona Clinic Liver Cancer staging system.

**Fig. 3:**
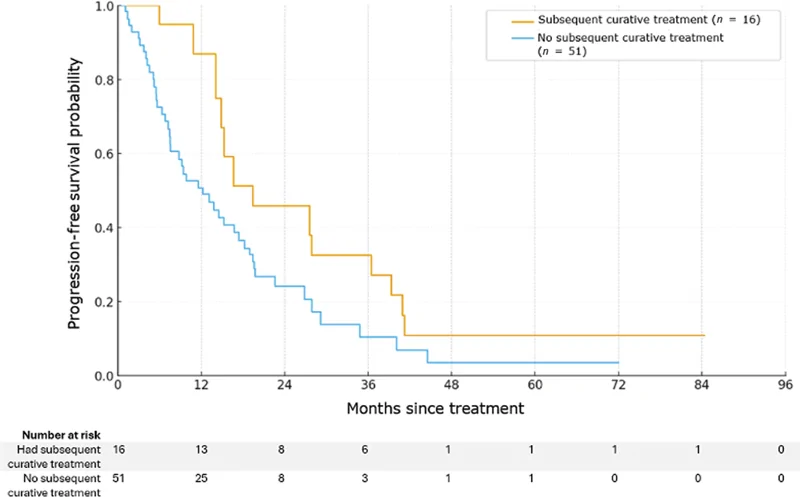
Kaplan–Meier curves of overall progression-free survival stratified by subsequent curative treatment.

Factors independently associated with shorter PFS were BCLC B/C ( Fig. 2 ) and the absence of subsequent curative treatment ( Fig. 3 ). Bilobar liver disease was significantly associated with poorer PFS on univariable analysis but did not retain statistical significance on multivariable analysis. Supplementary Table S2 (available in the online version only) in the Supplementary Appendix details the univariable Cox regression, while Table 2 details the multivariable Cox regression for overall PFS.

##### Overall Survival

All patients ( *n* = 68) were included in the OS analysis, with patients alive at last follow-up being censored. Median OS was 1,412 days (95% CI 596–NR), or 46.4 months. Thirty-four of 68 (50%) patients had died by the date of study closure, five patients (7%) were considered lost to follow-up, and 29 (43%) patients were presumed to be alive at study closure (i.e., no recorded death and with last follow-up within 6 months). Of these 29 presumed-alive patients, the earliest documented follow-up occurred 185 days before the study end date. Five patients had no known survival status in the electronic medical records and their last follow-up was 6 years before study closure, suggesting loss to follow-up.

Median OS for the 190 to 300 Gy group ( *n* = 44) was 699 days (95% CI 531–2244 days), or 23.0 months. For the >300 Gy group ( *n* = 24), median OS was 1,446 days (95% CI 796 days – NR), or 47.5 months. Observed differences in OS between these dose groups were not statistically significant.

**Fig. 4:**
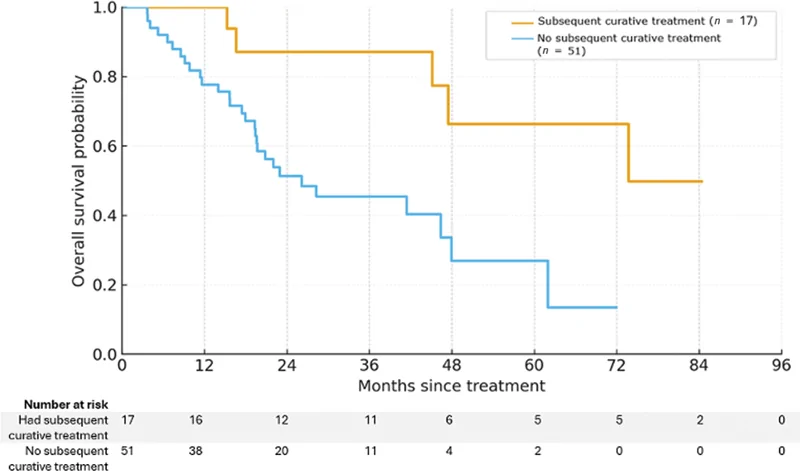
Kaplan–Meier curves of overall survival stratified by subsequent curative treatment.

Absence of subsequent curative treatment was independently associated with poorer OS ( Fig. 4 ). BCLC stage B/C and bilobar liver disease were significantly associated with poorer OS on univariable analysis but did not retain statistical significance in the multivariable analysis. Supplementary Table S3 (available in the online version only) in the Supplementary Appendix details the univariable Cox regression analysis, while Table 3 details the multivariable Cox regression analysis for OS.

To assess the potential confounding effect of subsequent curative interventions on OS, a sensitivity analysis was performed after excluding the 17 patients who underwent liver transplantation, surgical resection, or percutaneous ablation of their tumors, leaving 51 patients for analysis. In this sensitivity analysis, no variables, including TAD, were independently associated with OS on multivariable Cox regression ( Supplementary Table S4 , available in the online version only). These findings suggest that the observed lack of association between TAD and OS was not solely driven by patients who underwent subsequent curative treatment, although the reduced sample size limits definitive conclusions.

Median OS was not reached in the following subgroups: patients who underwent subsequent curative treatment, BCLC A, unilobar liver disease, Child-Pugh class A, solitary tumor, target tumor size <8 cm, no portal venous invasion, and no prior local treatment.

## Safety Outcomes

The type and number of adverse events likely related to the Y-90 treatments are detailed below ( Table 5 ). Six patients experienced treatment-related adverse events: two with possible radiation-induced liver disease, two with post-embolization syndrome, and two with radiation-induced peptic ulcer disease confirmed by endoscopic biopsy showing Y-90 microspheres. One patient's gastric ulcer required endoscopic hemostasis with noradrenaline injection and clip placement. The grade 2 to 3 laboratory abnormalities correspond to these six patients ( Table 5 ). All patients' liver function returned to baseline, either spontaneously or with a short course of steroids ( *n* = 2).

**Table 5:** Number of adverse events likely related to Y-90 treatment

Adverse event	Any-grade	Grade 1	Grade 2	Grade 3	Grade 4
*Clinical adverse events*					
Abdominal pain	10	7 ^b^ c,d	2 ^b^	1 e	
Bloating	1		1		
Dyspepsia	3	3			
Fever	3	3 ^d^			
Nausea/vomiting	5	4 ^d^	1		
Gastric ulcer	2	1 ^f^		1 e	
Ascites	2	2			
*Laboratory events*					
Alanine aminotransferase increased	7	4	2 a	1 ^d^	
Aspartate aminotransferase increased	10	7	1 a	1 c	1 ^d^
Alkaline phosphatase increased	6	3	2 c,d	1 ^b^	
Gamma-glutamyl transferase increased	5	1		4 a,b c,d	
Bilirubin raised	4	2	1 ^d^	1 a	
Anemia	1		1 e		
Platelet count decreased	1	1 ^d^			
Albumin decreased	4	3	1 ^d^		

Grade 2–4 adverse events were mostly experienced by the six patients a–f with:

a,b Post-embolization syndrome.

c,d REILD (patient d's baseline AST was elevated at 3.5x the upper limit of normal before Y-90 treatment).

e,f Gastric ulcer.

## Discussion

Glass and resin microspheres represent two distinct carriers for Y-90 SIRT, each with unique physical properties and clinical implications. Glass microspheres (e.g., TheraSphere) possess a much higher specific activity (approximately 2,500 Bq/sphere) compared to resin microspheres (e.g., SIR-Spheres, ∼50 Bq/sphere), allowing for the delivery of high absorbed doses with fewer particles. This makes glass microspheres particularly well-suited for radiation segmentectomy, where selective arterial infusion is used to achieve ablative doses to tumors confined to two to three segments of the liver. There has been robust evidence supporting the use of glass microspheres in this context, including prospective studies such as the LEGACY study, which demonstrated an 88.2% CR rate and 86.6% 3-year OS in early-stage HCC treated with glass Y-90 segmentectomy. 2 Similarly, the DOSISPHERE-01 trial showed significantly improved survival and response with personalized high-dose glass Y-90 (≥205 Gy) compared to standard dosing. 9 In contrast, while resin microspheres are more widely available and have been extensively used in intermediate-stage HCC, there has been a comparative paucity of evidence supporting their use in radiation segmentectomy.

A previous local study evaluating outcomes in patients with unresectable HCC treated with Y-90 resin microspheres found that a TAD at least 150 Gy yielded better OS (32.2 months vs. 17.5 months for those who received <150 Gy to tumor, *p* <0.001). 10 While TAD above 150 Gy was previously recommended for resin-based Y90 radiation segmentectomy, 11 more recently, there are emerging clinical and dosimetric evidence that higher doses should be used for ablative applications. In studies involving resin-based Y-90 segmentectomy, median TADs associated with favorable objective and CR rates ranged from 232 12 to 247 Gy. 13 Sarwar et al demonstrated, in a retrospective study of Y-90 resin microspheres radiation segmentectomy, lower disease progression at 2 years in the group that received ≥300 Gy than in the group that received <300 Gy (17 vs. 61%; *p* = 0.047). 12

Kokabi et al evaluated treatment response and toxicity in patients with treatment naïve HCC treated with resin microspheres and showed that complete and ORs can be predicted by Y90 TAD greater than 337 and 253 Gy, respectively, 14 and in a separate study evaluating personalized dosimetry in surgically unresectable HCC treated with resin Y-90 SIRT, found a mean TAD> 250 Gy resulted in prolonged mean OS and PFS. 15 Recent consensus guidelines recommend a dose of >250 Gy (via single compartment MIRD model) to be delivered when feasible. 11 13 14 16

We sought to evaluate our single-center experience for a dose–response relationship in the context of resin-based Y-90 SIRT with ablative intent. A TAD threshold of 190 Gy was selected based on the seminal explant analyses by Vouché et al, 4 which demonstrated a strong association between absorbed doses >190 Gy and CPN, albeit in the context of glass microspheres and radiation segmentectomy. This was subsequently supported by a multicenter explant analysis of 45 patients, 5 in which 86% of tumors receiving >190 Gy achieved CPN and all tumors receiving >400 Gy demonstrated CPN, as well as by the validation study by Toskich et al, 6 which showed increasing CPN rates with absorbed dose, reaching 75% at doses >500 Gy. Despite using these biologically and clinically plausible cutoffs, a statistically significant dose–outcome relationship was not observed in our cohort.

Although CPN thresholds have historically been derived from glass-microsphere explant datasets, emerging resin-microsphere explant data suggest that higher mean TADs (e.g., ≥433 Gy) 17 may be required to consistently achieve CPN, potentially explaining the absence of a clear dose–response relationship when applying a ≥190 Gy cutoff. In addition, restricting inclusion to patients receiving at least 190 Gy narrowed the evaluated dose range, effectively limiting analysis to a high-dose cohort, while the use of a dosimetry-only inclusion criterion resulted in a clinically heterogeneous, “all-comers” population in which inter-patient and inter-tumoral variability may have further attenuated observable dose–outcome associations.

The high degree of censoring resulted in relatively few observed events, limiting statistical power and potentially attenuating effect estimates. In particular, interpretation of the target TTP analysis warrants caution, as, only 17 out of 67 evaluable patients' target tumors demonstrated progression during follow-up; a substantial proportion of patients were censored at the time of subsequent local therapies or death prior to target tumor progression. This censoring may have been informative and could have inflated estimated time to progression in the lower-dose group, in contrast to the higher-dose group, where censoring for such reasons occurred in only about one-third of patients.

Our limited sample size likely reflects institutional practice patterns in years prior to the availability of stronger evidence supporting resin microspheres for radiation segmentectomy and dosimetry-guided dose escalation. Physician preference for glass microspheres in curative-intent segmentectomies may have been influenced by earlier evidence supporting their use in radiation segmentectomy and concerns that resin's lower specific activity—and correspondingly larger particle load—might heighten embolic risk in selective treatments. As a result, fewer patients receiving high-dose resin Y-90 were available for inclusion, limiting statistical power to detect dose–response relationships.

An important limitation of this study is the absence of post-treatment dosimetry, such that TAD was estimated from treatment planning rather than directly quantified from post-SIRT imaging. Consequently, discrepancies between planned and delivered dose could not be assessed and may have introduced misclassification of patients across absorbed-dose categories, possibly resulting in attenuation of dose–response relationships. Future dose–response analyses would be strengthened by incorporating post-treatment dosimetry using Y-90 Bremsstrahlung SPECT/CT or Y-90 PET/CT to more accurately quantify the delivered TAD. In an analogous study evaluating Y-90 resin radiation segmentectomy for HCC, 12 Sarwar et al found that actual TAD based on post-Y-90 treatment imaging was lower than the prescribed activity in 23 of 44 (52%) patients, highlighting the potential inaccuracy of relying solely on planned dosimetry. Variability in activity calibration timing may additionally influence microsphere-specific activity and actual tumor dose, highlighting the value of post-treatment dosimetric validation.

Although radiologic response was intended to be assessed at predefined 6- and 12-month intervals, the retrospective design and variability in real-world follow-up imaging resulted in accepted scans within a ± 2-month window, which may limit the generalizability of reported response and DCRs at these time points.

Notwithstanding these limitations, local disease control was substantial, with approximately 87 to 97% target tumor control within the first year after Y-90 SIRT and median target TTP not reached based on mRECIST criteria. These findings underscore the robust local tumor control achievable with Y-90 SIRT when a TAD of at least 190 Gy is delivered, while portal venous invasion remained independently associated with shorter target TTP. Our PFS and OS results also concur with prior studies 18 19 consistently showing superior long-term outcomes among patients who can be bridged or downstaged to curative-intent therapy, whereas patients who do not proceed to curative therapy generally experience poorer survival.

Across resin Y-90 studies, 12 15 20 median OS for HCC patients typically range from 23 to 26 months when personalized dosimetry is employed, with improved outcomes in cohorts treated at higher doses (mean OS of approximately 33.6 months when mean tumor dose >250 Gy 15 ). In our cohort of patients receiving at least 190 Gy, the median OS approached 46 months, though we acknowledge the potential confounder of subsequent therapies. Nevertheless, this pattern reflects real-world treatment pathways, where survival outcomes emerge from the combined influence of initial therapy and subsequent interventions.

The two cases of biopsy-confirmed radiation-induced peptic ulcer disease underscore the importance of preventive measures against non-target microsphere deposition during resin SIRT. Given the higher particle burden generally required with resin microspheres to deliver an equivalent radiation dose to that achieved with glass microspheres, meticulous angiographic assessment, careful catheter positioning, and vigilant attention to intraprocedural flow dynamics remain essential, particularly during high-dose resin SIRT delivery, to minimize the risk of extrahepatic microsphere deposition.

## Conclusion

Y-90 resin SIRT with a prescribed TAD of at least 190 Gy in HCC resulted in favorable 1-year target tumor disease control and a median OS of 46.4 months, while absence of subsequent curative treatment independently predicted inferior progression-free and OS. Among plausible reasons for the absence of a clear dose–response relationship, TADs substantially higher than 190 Gy may be required for consistent ablative efficacy. Prospective studies utilizing post-treatment dosimetry are needed to establish optimal absorbed-dose thresholds for ablative-intent Y-90 resin SIRT in HCC. Future research should also evaluate dose–response relationships in other hepatic malignancies, including colorectal and neuroendocrine liver metastases, to further inform personalized dosimetry and optimize treatment outcomes.
