# Supplementary material for: Real World Outcomes of Prescribed Ablative Dose Yttrium-90 Resin Selective Internal Radiation Therapy of Hepatocellular Carcinoma
Source: World J Nucl Med. 2026 Jul 15;25(3):197–207. doi: 10.1055/s-0046-1825760 (PMC13612124; doi:10.1055/s-0046-1825760)
Supplement: Supplementary file 1 [file 10-1055-s-0046-1825760-s2640010.pdf]

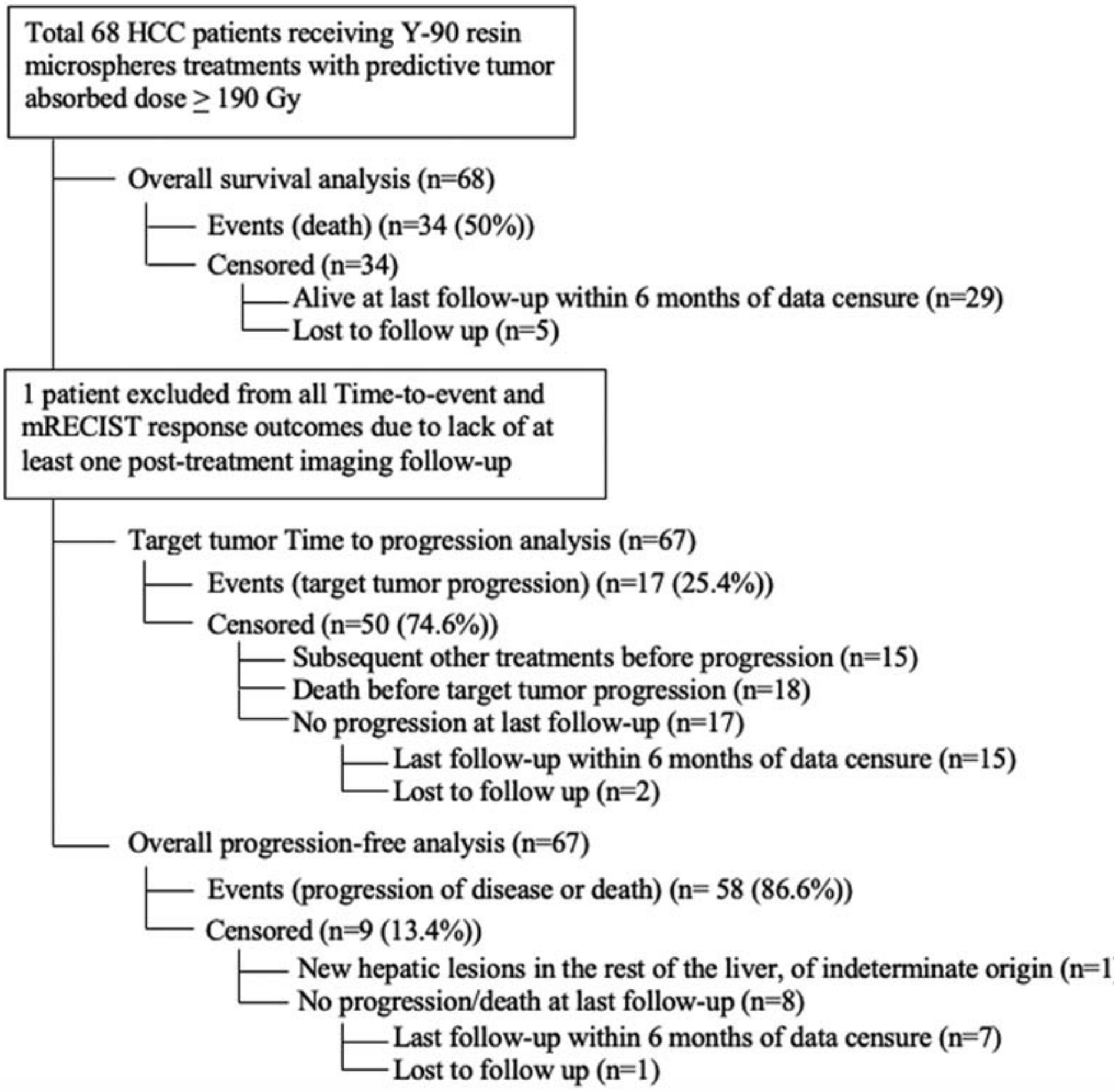

Supplementary Fig. S1 Study flow diagram.

Supplementary Table S1 Univariable Cox regression for target tumor time to progression

Variable	HR (95% CI)	*p*-Value
Size≥ 8 cm vs. size <8 cm	0.81 (0.22–2.96)	0.754
Portal vein invasion (PVI) vs. no PVI	5.06 (1.41–18.16)	0.013
No subsequent curative treatment vs. subsequent curative treatment	4.03 (0.53–30.91)	0.179
No previous local treatment vs. previous local treatment	0.81 (0.10–6.38)	0.838
TAD >300 Gy vs. TAD 190–300 Gy	1.55 (0.54–4.44)	0.416

Abbreviations: CI, confidence interval; HR, hazard ratio; TAD, tumor absorbed dose.

Supplementary Table S2 Univariable Cox regression for overall progression-free survival

Variable	HR (95% CI)	*p*-Value
Age >70 vs. age ≤70	0.78 (0.46–1.32)	0.351
Female vs. male	1.53 (0.85–2.75)	0.159
BCLC B/C vs. BCLC A	2.89 (1.61–5.19)	0.0004
Bilobar liver disease vs. unilobar disease	2.01 (1.01–3.97)	0.0455
No subsequent curative treatment vs. subsequent curative treatment	2.12 (1.11–3.95)	0.0216
TAD> 300 Gy vs. TAD 190–300 Gy	1.01 (0.59–1.73)	0.979

Abbreviations: BCLC, Barcelona Clinic Liver Cancer staging system; CI, confidence interval; HR, hazard ratio; TAD, tumor absorbed dose.

 © 2026. The Author(s).

Supplementary Table S3 Univariable Cox regression for overall survival

Variable	HR (95% CI)	*p*-Value
Age >70 vs. age ≤70	1.27 (0.62–2.58)	0.512
Female vs. male	1.22 (0.57–2.63)	0.606
BCLC B/C vs. BCLC A	2.84 (0.33–1.76)	0.004
Bilobar liver disease vs. unilobar disease	3.72 (0.49–2.14)	0.002
No subsequent curative treatment vs. subsequent curative treatment	4.20 (0.36–2.50)	0.009
TAD >300 Gy vs. TAD 190–300 Gy	0.61 (0.29–1.38)	0.203

Abbreviations: BCLC, Barcelona Clinic Liver Cancer staging system; CI, confidence interval; HR, hazard ratio; TAD, tumor absorbed dose.

Supplementary Table S4 Multivariable Cox regression for overall survival in patients without subsequent curative treatment

Variable	Adjusted HR (95% CI)	*p*-Value
Age >70 vs. Age ≤70	1.29 (0.54–3.04)	0.566
Female vs. male	1.93 (0.77–4.85)	0.161
BCLC B/C vs. BCLC A	2.14 (0.65–7.00)	0.208
TAD >300 Gy vs. TAD 190–300 Gy	0.52 (0.22–1.22)	0.133
Bilobar liver disease vs. unilobar disease	2.32 (0.79–6.82)	0.126

Abbreviations: BCLC, Barcelona Clinic Liver Cancer staging system; CI, confidence interval; HR, hazard ratio; TAD, tumor absorbed dose.

 © 2026. The Author(s).
